# Peptide-MHC-Based Nanomedicines for the Treatment of Autoimmunity: Engineering, Mechanisms, and Diseases

**DOI:** 10.3389/fimmu.2020.621774

**Published:** 2021-01-26

**Authors:** Pau Serra, Pere Santamaria

**Affiliations:** ^1^ Institut D’Investigacions Biomèdiques August Pi i Sunyer, Barcelona, Spain; ^2^ Julia McFarlane Diabetes Research Centre (JMDRC) and Department of Microbiology, Immunology and Infectious Diseases, Snyder Institute for Chronic Diseases and Hotchkiss Brain Institute, Cumming School of Medicine, University of Calgary, Calgary, AB, Canada

**Keywords:** peptide-major histocompatibility complex molecules, nanoparticles, autoimmune diseases, T-cell re-programming, T-regulatory type 1 cells

## Abstract

The development of autoimmunity results from a breakdown of immunoregulation and involves cellularly complex immune responses against broad repertoires of epitope specificities. As a result, selective targeting of specific effector autoreactive T- or B-cells is not a realistic therapeutic option for most autoimmune diseases. Induction of autoantigen-specific regulatory T-cells capable of effecting bystander (dominant), yet tissue-specific, immunoregulation has thus emerged as a preferred therapeutic alternative. We have shown that peptide-major histocompatibility complex (pMHC)-based nanomedicines can re-program cognate autoantigen-experienced T-cells into disease-suppressing regulatory T-cells, which in turn elicit the formation of complex regulatory cell networks capable of comprehensively suppressing organ-specific autoimmunity without impairing normal immunity. Here, we summarize the various pMHC-based nanomedicines and disease models tested to date, the engineering principles underpinning the pharmacodynamic and therapeutic potency of these compounds, and the underlying mechanisms of action.

## Introduction

The development of autoimmune disease results from dysregulated immune responses to self that are triggered by ill-defined environmental cues in genetically predisposed individuals. Such immune responses lead to the activation and recruitment of effector autoreactive T-cells into specific tissues/organs, the recruitment of additional inflammatory cell types to the site, chronic inflammation and, eventually, tissue/organ dysfunction and/or destruction. Given the autoantigenic complexity of most autoimmune disorders, targeting of effector autoreactive T-cell specificities is not a realistic therapeutic option for the treatment of these diseases. An alternative includes promoting the formation and/or expansion of regulatory autoreactive T-cell clonotypes capable of effecting bystander immunoregulation (against the many non-cognate autoantigenic epitopes that are targeted in the course of a specific disease). Several approaches that are potentially capable of eliciting bystander immunoregulation have been described over the last decade, but the mechanisms of action of some of these remain unclear and their therapeutic efficacy has not been thoroughly tested in non-contrived models of spontaneous, polyclonal autoimmunity [reviewed in (1)].

We have shown that profound and sustained ligation of antigen receptors on cognate effector autoreactive T-cells by nanoparticles (NPs) displaying multiple copies of disease-relevant peptide-MHC class I or class II complexes (pMHC-NP) can trigger their differentiation into regulatory T-cells *in vivo*. Upon pMHC-NP-induced expansion, these cognate, mono-specific autoreactive T-cells elicit self-sustaining regulatory cell networks that efficiently suppress polyclonal autoreactive T-cell responses in several murine models of autoimmunity, without compromising normal immunity. In this mini-review, we discuss the key engineering principles behind the pharmacodynamic activity of these compounds, the mechanisms underlying their therapeutic activity, and the disease models in which we have documented efficacy (**Table 1**).

**Table 1:** pMHC-based nanomedicines and models.

pMHC-nanomedicines	Disease target	Animal model	Disease tested	PD activity	Ther. activity	Reference
NRP-V7-K^d^-NP	T1D	NOD	TID	+	+	(2)
		B10.*H2^g7^*	None	−	−	(2)
		NOD.*G6pc2* ^K209A-F213A^	T1D	−	−	(2)
IGRP_206–214_-K^d^-NP	TID	NOD	TID	+	+	(2)
MimA2(DMK_138–146_)-D^b^-NP	TID	NOD	TID	+	+	(2)
TUM-K^d^-NP	None	NOD	TID	−	−	(2)
hIGRP_265–273_-A2K^b^-NP	TID	NOD.HHD	TID	+	+	(2)
INS_10–18_-A2K^b^-NP	TID	NOD.HHD	TID	+	+	(2)
Flu-MP_58-66_-A2K^b^-NP	None	NOD.HHD	TID	−	−	(2)
BDC2.5mi/IA^g7^-NP	TID	NOD	TID	+	+	(3)
		NOD *G6pc2* ^−/−^	TID	+	+	(3)
		NOD.*c3c4*	PBC	−	−	(4)
IGRP_4-22_/IA^g7^-NP	TID	NOD	TID	+	+	(3)
		NOD *G6pc2* ^−/−^	TID	−	−	(3)
IGRP_128-145_/IA^g7^-NP	TID	NOD	TID	+	+	(3)
HEL_14-22_/IA^g7^-NP	None	NOD	TID	−	−	(3)
GAD65_555(557I)–567_/DR4-NP	TID	hPBMC-NSG	TID	+	N/A	(3)
PPI_76–90(88S)_/DR4-NP	TID	hPBMC-NSG	TID	+	N/A	(3)
IGRP_13–25_/DR3-NP	TID	hPBMC-NSG	TID	+	N/A	(3)
pMOG_38–49_/IA^b^-NP	EAE	EAE in C57BL/6	EAE (pMOG_35–55_)	+	+	(3)
hPLP_175–192_/DR4-IE-NP	EAE	C57BL/6 *IAb^null^* *HLA-DR4-IE*	EAE (hPLP_175–192_)	+	+	(3)
hMOG_97–108_/DR4-IE-NP	EAE	C57BL/6 *IAb^null^* *HLA-DR4-IE*	EAE (hMOG_97–108_)	+	+	(3)
		C57BL/6 *IAb^null^* *HLA-DR4-IE*	EAE (hPLP_175–192_)	+	+	(3)
		C57BL/10.M *HLA-DR4-IE*	CIA	−	−	(3)
mCII_259–273_/DR4-IE-NP	CIA	C57BL/10.M *HLA-DR4-IE*	CIA	+	+	(3)
	CIA	C57BL/6 *IAb^null^* *HLA-DR4-IE*	EAE (hPLP_175–192_)	−	–	(3)
MOG_36–50_/IA^g7^-NPs	EAE	EAE in NOD	EAE (MOG_35-55_)	+	+	(4)
PDC_166–181_-IA^g7^-NPs	PBC	NOD.*c3c4*	PBC	+	+	(4)
		(NODxB6.*Ifng-ARE-Del* ^−/−^) F1	PBC	+	+	(4)
		Ad-FTCD-AIH in NOD	AIH	+	+	(4)
		Abcb4-KO (MDR3^−/−^)	PSC	+	+	(4)
		EAE in NOD	EAE (MOG_35-55_)	+	+	(4)
PDC_82–96_-IA^g7^-NPs	PBC	NOD.c3c4	PBC	+	+	(4)
FTCD_58–72_/IA^g7^-NPs	AIH	Ad-FTCD-AIH in NOD	AIH	+	+	(4)
CYPD_398–412_/IA^g7^-NPs	AIH	Ad-FTCD-AIH in NOD	AIH	+	+	(4)
		NOD.c3c4	PBC	+	+	(4)
		Abcb4-KO (MDR3^−/−^)	PSC	+	+	(4)
		EAE in NOD	EAE (MOG_35-55_)	+	+	(4)
hPDC-E2_122–135_/DRB4-NPs	PBC	hPBMC-NSG	PBC	+	N/A	(4)
hPDC-E2_249–262_/DRB4*0101-NPs	PBC	hPBMC-NSG	PBC	+	N/A	(4)
BDC2.5mi/IA^g7^-NP	T1D	NOD	T1D	+	N/A	(5)
		NOD.*RIP-hDTR* + DT	T1D	+	N/A	(5)
		EAE in NOD	EAE (MOG_35-55_)	+	−	(5)
		Ad-FTCD-AIH in NOD	AIH	+	−	(5)
		EAE+PBC in NOD.*c3c4*	EAE (MOG_35-55_) and PBC	−	− (PBC) − (EAE)	(5)
PDC_166–181_-IA^g7^-NPs	PBC	NOD	T1D	−	N/A	(5)
		NOD.*RIP-hDTR* + DT	T1D	+	N/A	(5)
		EAE in NOD	EAE (MOG_35-55_)	+	+	(5)
		EAE+PBC in NOD.*c3c4*	EAE (MOG_35-55_) and PBC	+	+ (PBC) − (EAE)	(5)
CYPD_398–412_/IA^g7^-NPs	AIH	NOD	T1D	−	N/A	(5)
		NOD.*RIP-hDTR* + DT	T1D	+	N/A	(5)
		EAE in NOD	EAE (MOG_35-55_)	+	+	(5)
		EAE+PBC in NOD.*c3c4*	EAE (MOG_35-55_) and PBC	+	+ (PBC) − (EAE)	(5)
MOG_36–50_/IA^g7^-NPs	EAE	NOD.*RIP-hDTR* + DT	T1D	−	N/A	(5)
		EAE in NOD	EAE (MOG_35-55_)	+	+	(5)
		EAE+ PBC in NOD.*c3c4*	EAE (MOG_35-55_) and PBC	+	− (PBC)− (EAE)	(5)
pMOG_38–49_/IA^b^-NP	EAE	EAE in C57BL/6	EAE (pMOG_35–55_)	+	+	(5)
	EAE	EAE+Ad-CYPD-AIH in C57BL/6	EAE (pMOG_35–55_) and AIH	+	+ (EAE)− (AIH)	(5)
	EAE	PDC_94–108_-IA^b^-NP-treated Ad-CYPD-AIH followed by EAE in C57BL/6	EAE (pMOG_35–55_) and AIH	+	+ (EAE) − (AIH)	(5)
Fla_462–472_/IA^b^-NPs	IBD	EAE+Ad-CYPD-AIH in C57BL/6	EAE (pMOG_35–55_) and AIH	−	− (EAE) − (AIH)	(5)
PDC_94–108_-IA^b^-NPs	PBC	EAE in C57BL/6	EAE (MOG_35-55_)	+	+	(5)
		EAE+Ad-CYPD-AIH in C57BL/6	EAE (pMOG_35–55_) and AIH	+	− (EAE) + (AIH)	(5)
	EAE	PDC_94–108_-IA^b^-NP-treated Ad-CYPD-AIH followed by EAE in C57BL/6	EAE (pMOG_35–55_) and AIH	+	+ (EAE) + (AIH)	(5)
CYPD_353–367_-IA^b^-NPs	AIH	EAE in C57BL/6	EAE (MOG_35-55_)	+	+	(5)
		EAE+Ad-CYPD-AIH in C57BL/6	EAE (pMOG_35–55_) and AIH	+	− (EAE) + (AIH)	(5)

TID, Type 1 Diabetes; EAE, Experimental Autoimmune Encephalomyelitis; CIA, Collagen-Induced Arthritis; PBC, Primary Biliary Cholangitis; AIH, Autoimmune Hepatitis; PSC, Primary Sclerosing Cholangitis.

## pMHCI-NPs as Triggers of Autoregulatory Memory-Like CD8+ T-Cell Expansion

Our initial attempts at developing an antigen-specific therapeutic approach for type 1 diabetes (T1D) aimed at triggering the deletion of a highly prevalent and diabetogenic CD8+ T-cell population specific for residues 206–214 of the islet-specific glucose-6-phosphatase catalytic subunit-related protein (IGRP_206–214_). This T-cell specificity plays a significant role in the progression of islet inflammation to beta cell destruction in NOD mice (6). Certain IGRP_206–214_ mimotopes could blunt disease progression in pre-diabetic mice by selectively triggering the deletion of high-avidity IGRP_206–214_-reactive clonotypes, while sparing their low-avidity counterparts (6). Surprisingly, treatment of pre-diabetic mice with the natural ligand or with super-agonistic mimotopes, which simultaneously deleted both high- and low-avidity clonotypes, was devoid of therapeutic activity (7). Subsequent experiments in T-cell receptor (TCR)-transgenic NOD mice expressing either low or high-affinity TCRs for IGRP_206–214_ demonstrated that the anti-diabetogenic effect of protective mimotopes was mediated by the low-avidity T-cell pool, which accumulated in the islets of Langerhans and presumably shielded beta cells from beta cell destruction by other autoreactive T-cell specificities (7, 8).

These observations exposed important limitations of mimotope-based immunotherapies. Namely, that complete deletion of individual mono-specific T-cell specificities is insufficient to blunt the progression of antigenically complex autoimmune disorders, and that the therapeutic success of antigen/peptide therapy hinges on the identification of optimal amino acid sequences, doses, and therapeutic regimens capable of eliciting the type of bystander regulation described above (9). Unfortunately, accurate prediction of the pharmacodynamic and therapeutic effects of specific peptide ligands *in vivo* is not currently possible or straightforward, thus hindering the translation of this approach for the treatment of human autoimmune disorders.

In an attempt to overcome these challenges, we sought to blunt the progression of T1D in NOD mice by simultaneously deleting multiple epitope T-cell specificities at once. We reasoned that, by virtue of their higher avidity for cognate T-cells and lack of co-stimulatory potential, NPs coated with multiple copies of disease-relevant pMHC class I (pMHCI) complexes, should be able to efficiently deplete cognate CD8+ clonotypes over a broad dose range. We further reasoned that, if this hypothesis were true, combinations of pMHCI-NPs targeting different CD8+ T-cell specificities should be able to substantially reduce the pool of beta cell killing effectors. Surprisingly, although treatment of NOD mice with the multi-specific pool of pMHCI-NPs had therapeutic effects, so did NPs exclusively displaying the IGRP_206–214_/K^d^ pMHCI (**Table 1**). Detailed examination of the therapeutic effects and mechanistic underpinnings of mono-specific pMHCI-NP therapy revealed that the therapeutic effect of these compounds was mediated by expansion of cognate low-avidity memory-like CD8+ T-cells with dominant regulatory potential. These memory-like autoregulatory CD8+ T-cells suppressed the activation of non-cognate autoreactive T-cell specificities by both suppressing and killing autoantigen-loaded professional antigen-presenting cells (APCs) in the pancreatic islets and pancreas-draining lymph nodes in an antigen-specific manner (2).

## Therapeutic Properties of pMHCII-NPs Displaying Tissue-Specific Epitopes

The allelic complexity MHC class I loci in humans limits the translational significance of pMHCI-NPs for human immunotherapy, as numerous compounds would need to be developed to treat a significant fraction of the patient population for any given autoimmune disease.

Our work with pMHCI-NPs suggested that treatment with these compounds harnesses a naturally-occurring negative feedback regulatory loop that might have arisen during natural evolution to oppose the progression of autoimmune inflammation. In turn, this idea suggested that such negative feedback regulatory loops might also exist in the autoreactive CD4+ T-cell compartment. This hypothesis predicted that treatment of autoimmune disease-affected mice with pMHCII-NPs would elicit the formation and/or expansion of autoantigen-specific regulatory CD4+ T-cells. Since there are strong associations between human autoimmune diseases and certain HLA class II types, and CD4+ T-cells play a central role in the initiation, progression and maintenance of most, if not all autoimmune diseases, we reasoned that these pMHCII-based compounds would have superior translational significance than their pMHCI-based counterparts.

We demonstrated that various murine T1D-relevant pMHCII-NPs (displaying BDC2.5mi/IA^g7^, IGRP_128–145_/IA^g7^ or IGRP_4–22_/IA^g7^) could stably restore normoglycemia in spontaneously diabetic NOD mice (**Table 1**). A similar outcome was obtained in wild-type C57BL/6 and HLA-DR4-transgenic C57BL/6 mice with experimental autoimmune encephalomyelitis (EAE, a murine model of multiple sclerosis). NPs displaying myelin oligodendrocyte glycoprotein (MOG)_38−49_/IA^b^ or human proteolipid protein (hPLP)_175–192_/DR4 complexes were able to reverse limb paralysis in these animals when administered at the peak of disease severity. Similar therapeutic effects were seen when using hMOG_97–108_/DR4-IE-NPs to treat hPLP_175–192_-induced EAE in HLA-DR4-transgenic C57BL/6 mice, demonstrating that the pMHCII displayed by these compounds need not have to target disease-initiating T-cells (**Table 1**). Likewise, NPs displaying mouse collagen II (mCII)_259–273_/DR4 could reverse both joint swelling and destruction in HLA-DR4-transgenic C57BL/10.M mice immunized with bovine collagen (3) (**Table 1**). These therapeutic effects were disease-specific because mCII_259–273_/DR4-NPs and hPLP_175–192_/DR4-NPs lacked therapeutic activity against EAE or collagen-induced arthritis, respectively, in HLA-DR4-transgenic mice (3) (**Table 1**). Furthermore, they did not compromise the ability of the host to clear a systemic viral infection or to mount antibody responses against an experimental vaccine (3).

## Pharmacodynamic Activity: pMHCII-NPs as Triggers of T-Regulatory Type 1 Cell Formation and Expansion

These therapeutic effects were invariably associated with systemic expansions of cognate CD4+ T-cells displaying a T-regulatory type 1 (Tr1)-like phenotype and transcriptional profile, as compared to murine Tr1-like cells described elsewhere (10). Experiments in diabetic NOD mice lacking expression of the antigenic epitope displayed on the pMHC-NP complex (hence lacking cognate epitope-experienced T-cells) demonstrated that the pharmacodynamic and therapeutic effects of both pMHCI- and pMHCII-NPs required the presence of autoantigen-experienced T-cells (2, 3) (**Table 1**). Clearly, these compounds target both naive and antigen-activated cognate CD4+ T-cells, but do so with divergent consequences. Thus, whereas pMHC-NPs induce activation-induced cell death of naive T-cells, they trigger the expansion of memory-like low avidity autoregulatory CD8+ cells (pMHCI-NP) or the differentiation of autoantigen-experienced effectors into Tr1 cells instead (pMHCII-NP). The lack of co-stimulatory signals on pMHCII-NPs, the absolute need for co-stimulation in the survival of naïve (albeit not memory) T-cells (11), coupled to the ability of repetitive antigen-specific stimulation of the TCR to elicit Tr1-like phenotypic features (12, 13) likely play a significant role in this outcome. Nevertheless, *in vitro* experiments have suggested that repetitive engagement of cognate TCRs by pMHCII-NPs, albeit necessary, is not sufficient to fully induce the differentiation of autoantigen-experienced cells into Tr1 cell progeny. It remains unclear if pMHCII-NPs can induce Tr1 cell formation from any cognate autoantigen-experienced CD4+ T-cell subset regardless of cell differentiation status, or only from a specific Tr1-poised precursor cell type. Ongoing transcriptional and epigenetic studies will shed light into the mechanistic underpinnings of this differentiation process.

## Key pMHC-NP Engineering Design Principles

The above observations prompted us to define what were the key pMHC-NP engineering design variables, to guide the development of next generation nanomedicines suitable for drug development and clinical translation. Extensive experimentation with various inorganic NP types (largely iron oxide-based) demonstrated that the biological activity of pMHC-NPs produced with NPs of a given size is a function of pMHC valency (number of pMHC monomers per NP) (14). *In vitro* studies using NPs of different sizes further indicated that the “optimal” pMHC valency values increased with NP size, indicating that pMHC density (number of pMHCs/surface area), rather than pMHC valency (absolute number of pMHCs/NP, regardless of NP size), is the most critical parameter (14). That is, it is not the absolute number of pMHC monomers per NP that determines potency, but rather the density of these pMHCs on the NP surface, such that NPs of different sizes carrying identical numbers of pMHCs will have different potencies. When tested on reporter Jurkat cells expressing cognate TCRs, these compounds lacked significant TCR triggering activity below a certain pMHC valency/density threshold. The TCR signaling potency of these compounds increased exponentially in response to relatively small increases in pMHC valency/density, starting at the pMHC valency/density threshold and ending at a “minimal optimal” pMHC valency/density value, at which the TCR signaling intensity plateaued. Substantial increases in pMHC valency/density above this minimal optimal valency did not result in significantly higher potency (14). These observations suggested that NPs displaying threshold and supra-threshold pMHC densities somehow promote cooperative TCR signaling.

**Figure 1:**
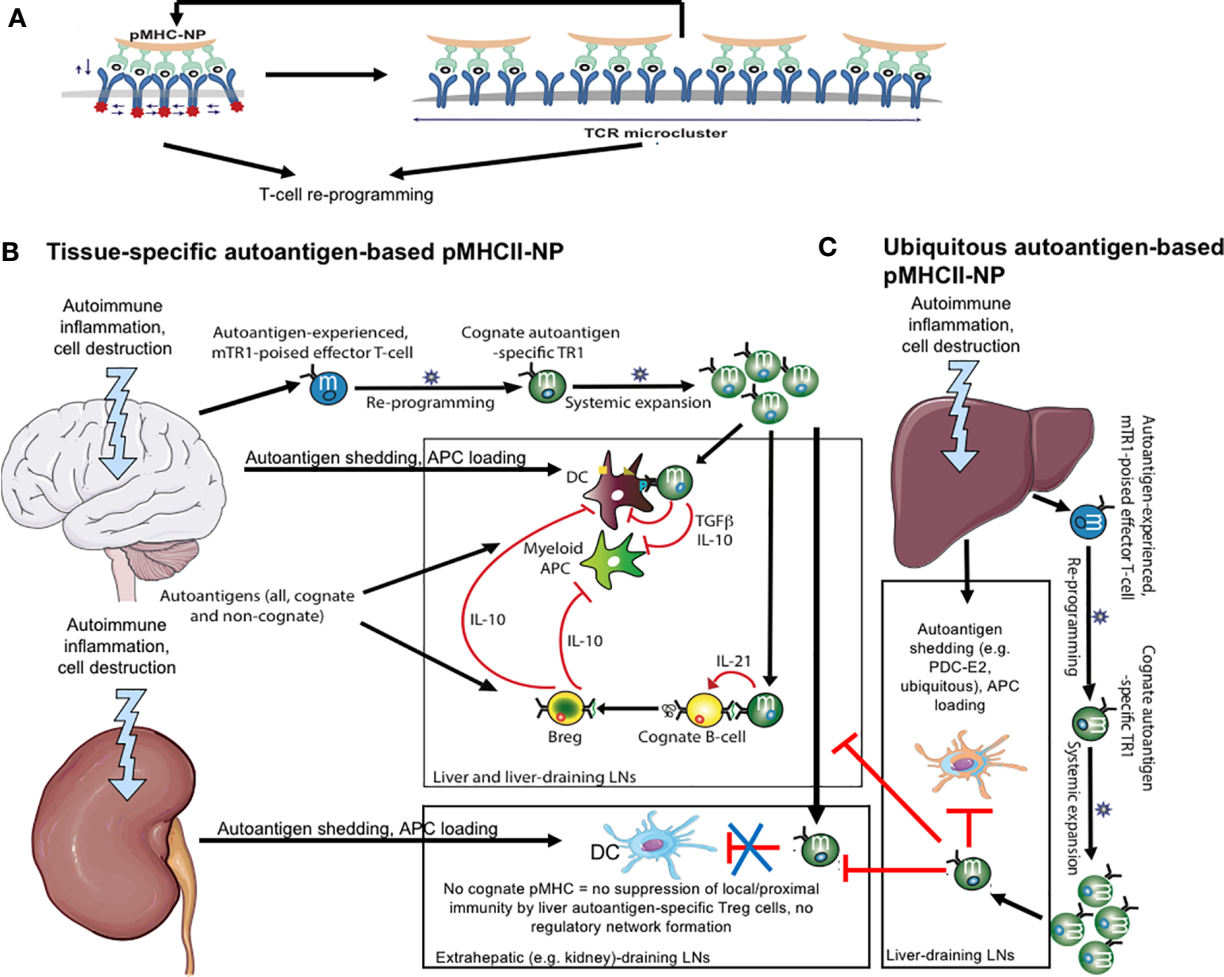
pMHCII-NPs: engineering and mechanisms. **(A)** pMHC-based nanomedicines function as antigen-receptor microclustering devices. Left, NP coated with a high-density array of mono-specific pMHC monomers elicits the simultaneous activation of multiple contiguous T-cell receptors, resulting in powerful signal amplification, cytoskeletal rearrangements, and TCR cluster formation. In turn, this increases the avidity of the target T-cell for additional incoming pMHC-NPs, further amplifying TCR signaling. Collectively, this profound, sustained and repetitive pMHC-NP engagement triggers T-cell re-programming through as yet unclear mechanisms. **(B)** pMHCII-based nanomedicines displaying epitopes from tissue-specific autoantigens [*e.g.* the central nervous system (CNS) in this cartoon] trigger the formation and subsequent expansion of CNS-specific T-regulatory type 1 (Tr1)-like cells. These cells biodistributed systemically, but exclusively undergo productive activation upon recognition of cognate pMHCII on professional APCs capable of delivering co-stimulatory signals (*i.e.* autoantigen-loaded DCs in the CNS or the CNS-draining lymph nodes). This elicits the local production of regulatory cytokines capable of suppressing autoantigen presentation to other autoreactive T-cell specificities. In addition, these cytokines recruit and locally re-program other immune cell types (*e.g.* B-cells) into cells with regulatory properties (Breg cells in this case). Collectively, these regulatory cell networks suppress local inflammatory processes, blunt disease progression and promote tissue repair. When these pMHCII-NP-induced CNS-specific Tr1 cells encounter APCs in other tissues/organs (*e.g.* kidney) lacking the Tr1 cells´ cognate autoantigen, they fail to engage the APC, hence to undergo productive activation. **(C)** pMHCII-based nanomedicines displaying epitopes from ubiquitous autoantigens (*e.g.* liver autoimmune disease-relevant) trigger the formation and subsequent expansion of Tr1-like cells that have the potential to suppress autoimmune responses in more than one tissue/organ.

*In vitro*, compounds displaying threshold and supra-threshold pMHC valencies/densities elicited very rapid (within 2h), vigorous and sustained (>24h) TCR signals, as compared to optimal concentrations of an agonistic CD3*ϵ* mAb or PMA/ionomycin, which triggered much slower responses that peaked at 14h and progressively decreased afterwards. Furthermore, imaging of pMHC-NP/T-cell interactions *via* transmission electron microscopy, super-resolution microscopy and scanning electron microscopy revealed that pMHC-NPs bind cognate T-cells as clusters of several NPs spanning ~100–150 nm that progressively grew to ~400 nm, culminating in internalization of the NPs in intracellular vesicles, starting ~3 h after binding. Importantly, cluster formation was only observed when using NPs coated at threshold and supra-threshold pMHC valencies/densities. Thus, pMHC-NPs function as sustained TCR nanocluster-binding and microcluster-triggering devices (**Figure 1A**). Collectively, these observations indicated that small NPs coated at the highest possible pMHC densities, allowing a near-perfect alignment of pMHCs on the NP and cognate TCRs on target T-cells, represent the most optimal design (14) (**Figure 1A**).

Subsequent *in vivo* experimentation with pMHCII-NPs suggested that whereas pMHC density regulates the efficiency of Tr1 cell formation, pMHC dose controls the magnitude of Tr1 cell expansion, indicating that pMHC density and pMHC dose have separate roles (14).

In terms of translation, the chemistry employed in the manufacture of iron oxide-based NPs is scalable. It is worth noting that, when used as MRI contrast agents in humans, these NP compounds are immunologically inert, biocompatible and safe. With regards to their pMHC-coated iron oxide NP counterparts, we have shown that such compounds have no off-target toxicity in zebrafish embryos, and do not cause hematological, biochemical or histological abnormalities in mice (14).

Our first generation pMHC compounds involved the expression of recombinant pMHC molecules in *E. coli* or *Drosophila* S2 cells followed by purification using 6xHis and/or streptag affinity chromatography. Low yields and the need to incorporate artificial affinity purification tags into the pMHC design represented significant obstacles for clinical translation. Expression in Chinese Hamster Ovary (CHO) cells and re-engineering of pMHC heterodimers as knob-into-hole-based Fc fusions addressed these limitations; KIH-based pMHC molecules are expressed at much higher yields than pMHCIIs heterodimerized using leucine zippers and can be purified to the desired levels of purity using protein A chromatography and additional polishing steps routinely used in the purification of biologics (15).

## Bystander Immunoregulation Mediated by Regulatory Cell Networks Arising Downstream of Tr1 Cell Formation

Studies in T1D (and later confirmed in other disease models) showed that pMHCII-NP-induced/expanded Tr1 cells suppressed the pro-inflammatory and antigen presentation capacities of local and proximal (*i.e.* in pancreas-draining lymph nodes) autoantigen-loaded dendritic cells (DCs) and myeloid APCs in an Interleukin-10 (IL-10)- and Tumor Growth Factor beta (TGF*β*)-dependent manner. Furthermore, recruitment of these antigen-specific Tr1 cells into the pancreas-draining lymph nodes of the treated mice promoted the formation/recruitment of interleukin-10 (IL-10)-producing CD1d^high^/CD5+ B-cells (**Figure 1B**). Transfer of cognate peptide-pulsed B-cells from donors expressing an IL-10 reporter transgene into treated recipients elicited *de novo* IL-10 expression in the donor B-cells, indicating that Breg cell formation in the pancreatic lymph nodes of these mice was induced by cognate Tr1-B-cell interactions. Antibody-mediated cytokine blockade demonstrated that, unlike APC suppression, Tr1-driven Breg cell formation was IL-21-dependent but IL-10 and TGFβ-independent. Both, BDC2.5mi/IA^g7^ tetramer+ T-cells and pancreatic lymph node-derived B-cells from treated donors could blunt the transfer of T1D to NOD.*scid* mice by splenocytes from untreated NOD mice, demonstrating the independent immunoregulatory activity of both cell types. Simultaneous transfer of both cell types had maximal (synergistic) therapeutic activity. Thus, pMHCII-NP therapy elicits the formation of disease-specific regulatory cell networks capable of restoring immune homeostasis.

## pMHCII-NPs Displaying Epitopes From Liver Autoimmune Disease-Relevant Ubiquitous Autoantigens

Autoimmunity in the liver manifests itself through various diseases, including primary biliary cholangitis (PBC), primary sclerosing cholangitis (PSC) and autoimmune hepatitis (AIH). In these diseases, unlike those discussed above, the autoimmune response recognizes ubiquitously expressed autoantigens, such as the mitochondrial pyruvate dehydrogenase complex-E2 component (PDC-E2) in PBC; or nuclear, cytoplasmic, or Golgi-enriched proteins, such as F-actin, formimidoyltransferase cyclodeaminase (FTCD), or cytochrome P450 (CYPD2D6) in AIH; or tropomyosin isoform 5 (hTM5) in PSC, among others. In addition, there is a significant subgroup of patients in which liver autoimmunity has features of both, cholestasis and autoimmune hepatitis, suggesting that autoimmune responses against certain autoantigenic targets in a given liver autoimmune disease (*e.g.* cholangitis) can spread to anatomic liver structures that are preferentially targeted in other liver autoimmune diseases (*e.g.* hepatitis). These observations begged the question of whether autoimmune liver disease-relevant pMHCII-NP compounds would be disease-specific (*e.g.* against PBC) or pan-liver autoimmune disease-specific (*e.g.* capable of blunting different liver autoimmune diseases).

Systemic delivery of two different PBC-relevant compounds (PDC-E2_166–181_/IA^g7^- and PDC-E2_82–96_/IA^g7^-NPs) blunted the progression of liver autoimmunity in NOD.*c3c4* mice and (NODxB6.*Ifng ARE-Del* ^−/−^) F1 mice, which spontaneously develop a form of liver autoimmunity that closely resembles human PBC (4) (**Table 1**). These compounds also blunted the progression of spontaneous PSC in *Abcb4* knockout mice and the progression of experimental AIH in NOD mice (induced by infection with an adenovirus encoding the human AIH-relevant autoantigen FTCD) (4) (**Table 1**). Likewise, both CYPD_398-412_/IA^g7^-NPs and PDC-E2_166-181_/IA^g7^-NPs (AIH and PBC-relevant nanomedicines, respectively) blunted Ad-hFTCD-induced AIH in NOD mice as efficiently as mFTCD_58-72_/IA^g7^-NPs. CYPD_398-412_/IA^g7^-NPs could also blunt the progression of PSC in NOD.*Abcb4* ^−/−^ mice (4) (**Table 1**). In these models, the various pMHCII-NPs suppressed disease by eliciting the formation and expansion of cognate Tr1-like CD4+ T-cells, the suppression of pro-inflammatory and antigen-presenting capacities of local and proximal APCs, and the formation/recruitment of Breg cells (4) (**Figure 1C**). Importantly, therapy with these compounds suppressed liver autoimmunity without impairing immunity against Influenza, Vaccinia, or *L. monocytogenes* infections or against allogeneic metastatic liver tumors (4).

Collectively, the above observations support the view that the tissue damage arising in response to a liver autoimmune disease initiated by autoreactive T-cells recognizing specific disease-relevant autoantigen(s) (*e.g.* PDC-E2 in PBC) results in the priming and recruitment of T-cell specificities targeting other autoantigens. Our work indicates that these secondary T-cell specificities can also be harnessed by pMHCII-NPs to blunt disease progression, as we had previously documented in EAE (**Table 1**). Thus, pMHCII-NPs need not have to target disease-initiating or prevalent autoreactive T-cell specificities to elicit therapeutic activity in a given autoimmune disease.

## Treatment of Extra-Hepatic Autoimmunity by pMHCII-NPs Displaying Ubiquitous Autoantigenic Epitopes

The above observations suggested that pMHCII-NPs displaying ubiquitously expressed epitopes might also have therapeutic activity against extra-hepatic autoimmune diseases. For these compounds to work, the epitopes derived from the ubiquitous protein would at least have to participate in the autoimmune response without necessarily playing a significant role in tissue destruction. Furthermore, the corresponding antigenic epitopes would have to be presented by professional APCs in amounts sufficient to trigger the activation of cognate CD4+ T-cells, to render them capable of responding to cognate pMHCII-NP treatment.

We sought to first investigate these assumptions by treating NOD mice with PDC-E2_166-181_/IA^g7^-NPs (PBC-relevant) and CYPD_398-412_/IA^g7^-NPs (AIH-relevant). Neither of these two compounds triggered the expansion of cognate Tr1-like CD4+ T-cells, suggesting that pancreatic beta cells either did not shed the corresponding antigenic epitopes, or did so in amounts insufficient to generate epitope-experienced CD4+ T-cells (**Table 1**). Diphtheria toxin (DT)-induced killing of ~50% beta cells of NOD mice expressing an X-chromosome-linked rat-insulin promoter-driven human diphtheria toxin receptor (hDTR) transgene rendered these mice responsive to PDC-E2_166–181_/IA^g7^-NPs, CYPD_398–412_/IA^g7^-NPs (PBC/AIH-relevant) and BDC2.5/IA^g7^-NPs (T1D-specific), but not MOG_36-50_/IA^g7^-NPs (EAE-specific, not expressed in pancreatic beta cells) (**Table 1**). The cognate pMHCII-NP-induced Tr1 cells that accumulated in the liver and pancreas-draining lymph nodes of these mice suppressed the activation of non-cognate beta-cell-autoreactive T-cells by local APCs, thus demonstrating that (1) pMHCII-NP-induced Tr1 cell formation requires autoantigen-experienced T-cells (2) that NOD mice harbor T-cells targeting ubiquitously-expressed antigens, and (3) that the priming of such cells requires antigen shedding (5) (**Figures 1B, C** **)**.

We then compared the ability of PDC-E2_166–181_/IA^g7^-NPs and CYPD_398–412_/IA^g7^-NPs (*vs.* BDC2.5/IA^g7^-NPs and MOG_36–50_/IA^g7^-NPs as negative and positive controls, respectively), or PDC-E2_94–108_/IA^b^-NPs and CYPD_353–367_/IA^b^-NPs to blunt MOG_36–55_-induced EAE in NOD and C57BL/6 mice, respectively. These experiments indicated that, upon oligodendrocyte damage, both PDC-E2 and CYPD2D6 (but not BDC2.5, which is not expressed in the CNS) are delivered to proximal APCs for autoreactive CD4+ T-cell priming, enabling Tr1 cell generation by cognate pMHCII-NPs, their recruitment to the CLNs, and suppression of EAE (**Table 1**).

Subsequent experiments using MOG_38–49_/IA^b^-, PDC-E2_94–108_/IA^b^-, CYPD_353–367_/IA^b^- and Fla_462–472_/IA^b^-NPs (as a negative control) in B6 mice having Ad-hFTCD-induced AIH and/or EAE revealed that Tr1 cell recruitment and therapeutic effects require local autoantigen expression (**Figure 1B**). Interestingly, liver inflammation in mice simultaneously having both EAE and AIH sequestered the ubiquitous antigen-specific Tr1 cells away from the CNS, abrogating their ability to blunt CNS autoimmunity (**Table 1**). Resolution of liver inflammation released these cells for recruitment to the CLNs, enabling them to blunt EAE (5) (**Table 1**) (**Figure 1C**). When we superimposed EAE onto the more aggressive, chronic form of liver autoimmunity that develops in NOD.*c3c4* mice, the three pMHCII-NPs tested (PDC-E2_166–181_/IA^g7^-, CYPD_398–412_/IA^g7^- and MOG_36–50_/IA^g7^-NPs) had pharmacodynamic activity but lacked therapeutic activity; liver inflammation in these mice retained antigen-specific Tr1 cells non-specifically (5) (**Table 1**).

To better understand how pMHCII-NP-expanded Tr1 cells traffic to multiple sites of inflammation (in co-morbid mice), we developed a mathematical model composed of a system of nonlinear ordinary differential equations (16). We compartmentalized the model into separate cell pools, each representing the organs under consideration, and evaluated the validity of the above experimental observations to understand the interplay between Tr1-cell allocation and pMHCII-NP therapeutic efficacy. In agreement with the experimental data, this model suggested that cognate autoantigen expression and local Tr1-cell retention are key determinants of effective regulatory-cell function downstream of pMHCII-NP therapy. Tissues competing for the same Tr1 resource (*i.e.* in co-morbid mice) may give rise to competitive autoimmunity where neither tissue will recruit a sufficient number of Tr1 cells beyond the suppression threshold (due to either impaired recruitment/retention or inefficient Tr1 suppressive potential) (16).

Collectively, these data indicated that (1) autoreactive T-cells targeting ubiquitous antigens can be awakened by antigen shedding from different cells/tissues (**Figure 1C**); (2) local autoantigen expression is required for the regulatory activity of antigen-specific Tr1-like cells (**Figures 1B, C** **)**: (3) liver inflammation has the potential to non-specifically draw T-regulatory cells away from sites of cognate autoantigen expression and autoimmune inflammation.

## Pharmacodynamic Activity of Human Autoimmune Disease-Relevant pMHCII-NPs in Humanized Mice

The pharmacodynamic activity of murine pMHCII-NPs in mice could be replicated in NOD.*scid/Il2rg* ^−/−^ (NSG) mice engrafted with peripheral blood mononuclear cells (PBMCs) from patients. Treatment of NSG mice humanized with PBMCs from DRB1*0301+ and/or DRB1*0401+ T1D patients with NPs displaying human IGRP_13–25_-DRB1*0301 or human pre-proinsulin (PPI)_76-90_/DRB1*0401 complexes resulted in the expansion of cognate IL-10-producing CD4+ T-cells co-expressing the Tr1 cell markers CD49b and LAG-3 (**Table 1**). Similar observations were made in NSG hosts reconstituted with PBMCs from DRB4*0101+ or DRB1*0801+ PBC patients in response to treatment with PDC-E2_122-135_/DRB4*0101-, PDC-E2_249-262_/DRB4*0101-, and PDC-E2_629–643_/DRB1*0801-NPs (**Table 1**). These observations support the translational potential of these compounds for the treatment of human autoimmunity, and introduce a preclinical validation tool for human pMHCII-NP candidates. Further refinement of this model, including the use of mice with a more developed humanized peripheral immune system, will further facilitate the pre-clinical evaluation of clinical candidates.

## Concluding Remarks

The therapeutic activity of NPs coated with disease-relevant pMHC molecules was an accident of curiosity-driven research that suggested that these compounds, initially designed for T-cell deletional therapy, could elicit bystander immunoregulation. Studies on a significant number of spontaneous and experimental autoimmune disease models using numerous pMHC-NP compounds have established the therapeutic potential of this approach to treat a whole host of autoimmune disorders in a disease-specific manner without compromising normal immunity. We have defined the key NP and pMHC engineering principles that are responsible for pharmacodynamic activity and have dissected mechanisms underlying therapeutic activity, namely Tr1 cell formation from an antigen-experienced precursor type, followed by systemic expansion, recruitment to the target tissue and formation of regulatory cell networks responsible for sustained and comprehensive therapeutic activity. The composition of these regulatory cell networks and the molecular cues responsible for their assembly and homeostasis likely vary as a function of disease type and organ. In liver autoimmunity, for example, the antigen-specific Tr1 cells and Breg cells that arise in response to pMHC-NP therapy cooperatively induce the recruitment and re-programming of neutrophils into a regulatory cell subset that resembles granulocyte myeloid-derived suppressor cells (MDSCs). The nature of the autoantigen-experienced T-cell type that gives rise to cognate Tr1 cells in response to pMHCII-NP therapy remains unclear, and so do the mechanisms *via* which sustained TCR signaling re-programs this precursor cell type into a Tr1 cell type.

The ability of these compounds to suppress autoantigen-loading APCs may explain why they spare normal immune responses to pathogens. The short half-life of dendritic cells *in vivo* implies that *de novo* suppression of newly recruited (non-autoantigen-loaded) APCs is required for sustained immunoregulation. On the other hand, this allows new, non-immunosuppressed APCs to process and present pathogen-derived antigens to pathogen-specific T-cell specificities. In addition, during a local infection, antigens derived from local pathogens likely overwhelm the APCs’ antigen presentation machinery, diluting expression of the Tr1’s cognate pMHC below the threshold required for Tr1 cell-induced APC immunoregulation. Upon clearance of the infection, new incoming APCs would then regain the ability to present the Tr1’s cognate pMHC, allowing these Tr1 cells to resume their anti-inflammatory activity.

At the translational level, we have made significant progress in candidate pMHCII identification and selection for specific autoimmune diseases. Our experimental work in mice has suggested that most, if not all, CD4+ T-cell specificities recognizing autoantigenic epitopes expressed by the target tissue of a given autoimmune disease can be re-programmed into Tr1 cells *in vivo via* pMHCII-NP therapy. Ideal clinical candidates are those displaying epitopes from prevalent tissue-specific autoantigens in the context of allelic MHCII types expressed by a significant fraction of the patient population. For autoimmune diseases with strong HLA class II associations, such as T1D or Celiac Disease, the choice of HLA type is straightforward. For diseases in which there is not a strong HLA class II allelic bias, the use of MHCII molecules encoded in oligomorphic HLA class II loci, such as DRB3, DRB4 and DRB5 loci is desirable. Up to three different pMHC-NP compounds would be sufficient to treat >80% of the patient population for any given autoimmune disorder. Notwithstanding the progress to date, candidate pMHCII selection remains a bottleneck that would benefit from the availability of improved, higher throughput methods capable of enumerating the frequency of defined pHLAII specificities (as opposed to peptide specificities regardless of HLA restriction) in patients’ peripheral blood samples. Although we have provided compelling evidence supporting translational potential, we do not yet know whether these compounds will be effective in clinical trials.

Lastly, the work done to date begs the question of whether T-cell types other than CD8+ and CD4+ T-cells, such as invariant Natural Killer T-cells or Mucosal Associated Invariant T-cells, can also be re-programmed into autoimmune disease-suppressing cell types using MHC-based nanomedicines. The next few years should provide answers to these outstanding questions.

## Author Contributions

The manuscript was written and edited by both co-authors. All authors contributed to the article and approved the submitted version.

## Funding

The authors’ work summarized here was funded by the Canadian Institutes of Health Research (CIHR), Diabetes Canada, the Crohn’s and Colitis Foundation of Canada, the Multiple Sclerosis Society of Canada (MSSC), ISCIII and FEDER (PIE14/00027, PI15/0797), REEM (Red Española de Esclerosis Múltiple), NEURON-ERANET (European Research Projects on Neuroinflammation; NEURON7-FP-715-018), the Praespero Foundation, the Ministerio de Economia y Competitividad of Spain (MINECO; RD16/0015/0020), and Generalitat de Catalunya (SGR and CERCA Programmes). The JMDRC is supported by Diabetes Canada.

## Conflict of Interest

PS is the scientific founder of Parvus Therapeutics Inc. and has a financial interest in the company.

The remaining author declares that the research was conducted in the absence of any commercial or financial relationships that could be construed as a potential conflict of interest.
